# Relationship between Sensory Attributes, (Dis) Liking and Volatile Organic Composition of Gorgonzola PDO Cheese

**DOI:** 10.3390/foods10112791

**Published:** 2021-11-12

**Authors:** Luisa Torri, Eugenio Aprea, Maria Piochi, Giorgia Cabrino, Isabella Endrizzi, Alessia Colaianni, Flavia Gasperi

**Affiliations:** 1 University of Gastronomic Sciences, Piazza Vittorio Emanuele II 9, 12042 Pollenzo, Italy; m.piochi@unisg.it (M.P.); g.cabrino@unisg.it (G.C.); alessia.colaianni31@gmail.com (A.C.); 2 Research and Innovation Centre, Fondazione Edmund Mach, Via Mach 1, 38010 S. Michele all’Adige, Italy; eugenio.aprea@fmach.it (E.A.); isabella.endrizzi@fmach.it (I.E.); flavia.gasperi@fmach.it (F.G.); 3 Center Agriculture Food Environment C3A, University of Trento/Fondazione Edmund Mach, Via Mach 1, 38010 S. Michele all’Adige, Italy

**Keywords:** blue-veined cheese, drivers of liking, soapy flavor, rate-all-that-apply test, volatile organic compounds, SPME/GC-MS, orthogonal partial least squares regression

## Abstract

Blue-veined cheese tends to polarize the consumers’ affective responses due to its strong flavor. This study aims to: (i) explore the consumers’ sensory perceptions and liking of Gorgonzola PDO cheese; (ii) identify the sensory drivers of acceptance for Gorgonzola in the function of the cheese style; (iii) characterize them by the volatile organic compounds (VOCs); and (iv) explore the relationships of the VOCs with sensory perception and liking. Six samples of Gorgonzola cheese differing in style (sweet vs. piquant), aging time (70–95 days), and production process (artisanal vs. industrial) were evaluated by 358 subjects (46% males, 18–77 years) using liking and Rate-All-That-Apply (RATA) tests. The cheese VOCs were measured by SPME/GC-MS. Liking was significantly higher for the sweet cheese than for the piquant cheese and for the artisanal cheese than for the industrial samples. Penalty Analysis showed that ‘creamy’, ‘sweet’, ‘nutty’, and ‘salty’ were significant drivers of liking while the ‘soapy’ and ‘ammonia’ flavors turned out to be drivers of disliking. Fifty-three VOCs were identified. Regression models revealed the significant highest associations between the VOCs and ‘ammonia’, ‘pungent’, ‘soapy’, and ‘moldy’ flavors. A good association was also found with the consumers’ liking. The identification of the sensory drivers of (dis) liking and their relationship with the VOCs of Gorgonzola opens up a new understanding of the consumers’ blue-veined cheese preferences.

## 1. Introduction

Gorgonzola is an Italian blue-veined cheese, produced with full-fat pasteurized cow’s milk and recognized as a Protected Designation of Origin (PDO) by the European Union [1,2,3]. In the field of Italian PDO cheese in the world, Gorgonzola PDO is once again the third cow’s milk cheese by produced volume, after Grana Padano and Parmigiano Reggiano [4]. Its production is limited to some provinces in the regions of Lombardy and Piedmont in Italy [5]. Depending on the process parameters (especially the aging time), two different Gorgonzola styles are available on the market: a traditional variety named ‘Piccante’ (Piquant), and a ‘Dolce’ style (Sweet) with a more delicate taste and a less pungent flavor [6]. The Gorgonzola is included among the so-called ‘blue-veined cheeses’ (with other types of cheese, such as the French Roquefort, the English Stilton, the Danish Danablu, etc.), produced by the inoculum of mold spores such as *Penicillium roqueforti* [7,8], and it is characterized by the typical piercing (in Italian ‘siringatura’) of the cheese before the aging phase. The presence of blue molds in cheese paste gives this group of various types of cheese a peculiar appearance (with the typical blue-greenish veins), and the high biochemical activities of the enzymes of these molds produce the typical aromas and colors, ranging from white to straw-yellow, and the different textures, varying from soft to more compact [9]. The flavor ranges from mild to sharp, depending on the age and the cheese type. The Gorgonzola revealed a quite different composition from the other blue-veined types of cheese as this cheese has an extensive degradation of both αs1- and β-caseins; it contained considerably higher concentrations of small peptides and free amino acids [10], and it has a high final pH (>6.0) with an approximate composition of about 30% fat, 20% protein, and 45% moisture [11].

From a commercial point of view, blue-veined cheeses seem to polarize the consumer preferences of those who like them and those who dislike them. Although some researchers have explored consumers’ liking and preferences for blue-veined cheese [12,13], only very few have evaluated Gorgonzola. A couple of studies were focused on the effect of cheese combination on the hedonic acceptability of cheese-beverage pairings [14,15], without deepening the understanding of the preferences for this product. A study showed that the liking for Gorgonzola hugely varies among children, with clusters liking it very much and clusters extremely disliking it [16]. In general, the reasons for not liking a particular type of cheese may be due to low familiarity with it [17]. However, Gorgonzola did not show an increase in liking among non-familiar consumers (Korean) after repeated exposure, in contrast to what was observed for the other four different types of cheese [18], suggesting that some consumers may not overcome some of the peculiar sensory properties of Gorgonzola, even through exposure.

Despite the sensory profiles of the Gorgonzola cheese samples obtained by trained assessors [14] or experts [19] and reported in the literature, the perception of the sensory attributes of Gorgonzola by consumers, or that which concerns the drivers of preference for this cheese, are poorly described. Thus, the reasons for (dis) liking Gorgonzola have not been systematically explored yet. Due to the high economic value represented by Gorgonzola and blue-veined cheese and the lack of specific data on negative and positive sensory drivers for their acceptability, it would be advisable to investigate this topic further.

Some previous studies focused on the relationships between the sensory aspects and the chemical composition of blue-veined cheese [20,21].

To characterize and quantify the molecules contributing to the cheese flavor, several instrumental techniques can be applied. For example, among the most common are the continuous liquid–liquid extraction combined with high-resolution gas chromatography (HRGC); the HRGC combined with mass spectrometry (MS) and HRGC-olfactometry [6]; the solvent extraction GC-MS; the headspace solid-phase-micro-extraction technique (SPME/GC–MS); and the direct headspace analysis (APCI–MS) [22] were used. As the analysis of volatile organic compounds (VOCs) in cheese has deserved great attention among researchers, the available instrumental techniques have been recently reviewed [23]. Other techniques were used to quantify non-volatile molecules in Gorgonzola, such as the chromatographic method HPAEC-PAD for sugar quantification that is responsible for the different degrees of sweetness and spiciness [24].

To the best of the authors’ knowledge, no paper has specifically focused on exploring the sensory drivers for the Gorgonzola (dis) liking, nor is there a paper that has associated the specific sensory properties with the volatile composition of Gorgonzola. Thus, the aims of this research were: (i) to explore the consumers’ sensory perception of sweet and piquant Gorgonzola cheese; (ii) to identify the positive and negative sensory drivers of acceptance for Gorgonzola cheese; (iii) to characterize the VOCs in Gorgonzola cheese by HS-SPME/GC-MS; and (iv) to explore the possible relationships of the VOCs with the consumers’ sensory perception and liking.

## 2. Materials and Methods

### 2.1. Gorgonzola PDO Cheese

Six Gorgonzola PDO cheese products varying for style (sweet vs. piquant), aging time (70–95 days) and production process (artisanal vs. industrial) (Table 1) were chosen to represent the variability of the Gorgonzola cheese on the market. All the cheese products were produced during the summer of 2019 by IGOR s.r.l. (Novara, Italy), one of the main Gorgonzola cheese producers in the PDO production area. The artisanal production was characterized by a discontinuous process and the manual execution of some operations by an operator (e.g., addition of the raw materials, curd cutting, and placing the curd in the molds), while the industrial production process was continuous and fully automatized, without any manual intervention by an operator. The cheese wheels had an approximate weight of 11–12 kg each. For each cheese sample, six pieces of 1.5 kg (corresponding to 1/8 of the cheese wheel) from six different cheese wheels of the same production batch were tested (9 kg in total for each cheese sample; overall, 54 kg of cheese). All the cheese products were made with whole cow’s milk (98.26%), selected milk enzymes (1.5%), selected *Penicillium* molds (0.01%; different strains of *Penicillium roquefortii* used for the different cheese products), dehydrated sea salt (0.21%), and animal rennet (0.02%).

Prior to the analysis, all the products were stored at 4 °C. Before the sensory test, the outer rind (1 cm) of each 1/8 of a cheese wheel was discarded, and the products were cut into 15 g parallelepipeds. At the same time, three different portions were sampled from each 1/8 of a cheese wheel and placed (no headspace left) in a 96 mL plastic cup that was then hermetically sealed and stored at −20 °C until the volatile compound determination.

### 2.2. Sensory Evaluation

#### 2.2.1. Participants

The study involved 358 subjects (54% females; age range: 18–77 years; average age: 41; standard deviation: 15; 71% Italians). The participants were recruited by email, websites, Facebook, and articles published in local newspapers.

#### 2.2.2. Evaluation Procedure

For the sensory tests, the cheese products (15 g parallelepipeds) were presented in hermetically sealed disposable plastic containers (96 mL), codified with three-digit random codes, in a randomized and balanced order, and at room temperature (20 °C). All the cheese products were analyzed over four consecutive days when edition XII of the international cheese exhibition, organized by the Slow Food movement and called ‘Cheese’, took place in 2019 (Bra, Italy). The consumers’ evaluation included: (1) a liking test; (2) a Rate-All-That-Apply (RATA) test; and (3) a short questionnaire on socio-demographic data and habits regarding the consumption of blue-veined cheese.

Firstly, the overall liking for each cheese sample was rated by consumers on the Labeled Affective Magnitude (LAM) scale, ranging from 0 (the greatest imaginable dislike) to 100 (the greatest imaginable like) [25]. As the second task, the participants performed a RATA test [26] with a list of 12 sensory attributes related to taste (salty, sweet), flavor (ammonia, cooked vegetables, floral, moldy, nutty, soapy, toasted), and mouthfeel (creamy, grainy, pungent). The list of descriptors was developed taking inspiration from the ‘Cheese aroma wheel’ [27] and from a list that was informally pre-tested to assess the appropriateness of the attribute list. For each product and each subject, the sequence of the attributes in the list was randomized following a design to balance the presentation order [26]. A list of attribute definitions was not provided; thus, each subject was required to interpret the meaning of the attributes by him/herself. Each consumer selected all the attributes perceived in the sample. Once the subject had chosen an attribute, a generalized Labelled Magnitude Scale (gLMS; 0 = no sensation, 100 = the strongest imaginable sensation of any kind) [28] became available on the PC screen and participants rated the perceived intensity of the selected attribute. Prior to the tasting, the subjects were extensively instructed in the use of the scale. ‘The strongest imaginable sensation of any kind’ was defined as the most intense sensation that involves remembered/imagined sensations in any sensory modality, including non-oral sensations, such as loudness, oral pain/irritation, or sight (e.g., the loudest sound ever heard, the most intense pain experienced, or the brightest light ever seen) [29,30]. Between products, a rinsing procedure was inserted.

The questionnaire aimed to collect information about age, sex, nationality, smoking attitude (smoker, ex-smoker, never-smoker), weight and height, and the frequency of consumption of blue-veined cheese (less than three times a year; less than once a month; 1–3 times a month; once a week; at least 2–3 times a week). Moreover, consumers declared their preference between the sweet and the piquant style of Gorgonzola Cheese.

The subjects were asked not to eat, drink, smoke, or wear perfume or lipstick for 1 h before the evaluation session. The participants took approximately 35–40 min to complete their tasks. The tests were performed in computerized individual sensory booths, neutralized from any contaminant odors, under white light. The data were collected with the software Fizz ver. 2. 47 B (Biosystèmes, Couternon, France).

### 2.3. Volatile Organic Composition

Thawed cheese (about 40 g taken from the six products prepared before the sensory test, as described in 2.1, and stored at −20 °C until the day before) was homogenized for 2 min with distilled water in the proportion 3:4 (three parts of cheese and four parts of water, on a weight basis) using Ultra Turrax. Then, a 7 g sample was introduced in a 20 mL glass vial, spiked with 50 μL of a solution of 4-methyl-2-pentanone (Aldrich, Milan, Italy) used as an internal standard (IS). The volatile compounds were measured according to Bottiroli et al. [31] with a slight modification of the temperature program. In brief, after 30 min of equilibration at 40 °C, the SPME fiber (2 cm, DVB-Carboxen–PDMS, SUPELCO, Bellefonte, PA, USA) was inserted in the headspace of the vial for 30 min and then desorbed at 250 °C in the injector port of a GC interfaced with a mass detector operating in an electron ionization mode (EI: 70 eV) (GC Clarus 500, PerkinElmer, Norwalk, CT, USA). Mass spectra were acquired in the scan range from m/z 33 to 300. The procedure was automatically managed by an auto-sampler (CTC combiPAL, CTC Analysis AG, Zwingen, Switzerland). Separation was achieved on an HP-Innowax fused-silica capillary column (30 m, 0.32 mm inner diameter, 0.5 μm film thickness; Agilent Technologies, Palo Alto, CA, USA). The temperature program was set progressively as follows: 40 °C for 3 min, 180 °C for 6 min at 4 °C min^−1^, and 220 °C for 3 min at 5 °C min^−1^. The total run was 55 min. Helium was used as the carrier gas at a flow rate of 2 mL/min. The transfer line temperature was kept at 220 °C.

Linear retention indices (LRI) were calculated under the same chromatographic conditions, injecting C7-C30 n-alkane series (Supelco, Bellefonte, PA, USA). The compounds were identified using the mass spectra matching the NIST-2014/Wiley 7.0 libraries and by comparing the calculated LRI with those available from the literature. The compound concentrations were reported as the equivalent of the IS.

A total of 36 cheese samples were analyzed: 6 samples per each of the six Gorgonzola cheese products (obtained by sampling from six different pieces, see Section 2.1). For each sample, four technical replicates (four different vials filled with the same cheese sample) were considered, and the data were reported as the average values of the four technical replicates.

### 2.4. Statistical Analysis

#### 2.4.1. Sensory Data

A two-way analysis of variance (ANOVA) model to estimate the effect of the Gorgonzola cheese style (sweet vs. piquant) and the production process (artisanal vs. industrial) on overall liking was performed. Three two-way ANOVA mixed models with the subjects as the random factor.were performed to estimate the effect of the product (six levels) as a fixed factor of liking in three groups of subjects: all males and females, respectively. In order to investigate the gender effect on the mean liking of all six products and of each cheese, separate *t*-tests were applied. Significant mean values in the ANOVA models were followed by the Tukey’s HSD comparison test or the Gomes-Howell test, accounting for unequal sample size when appropriate.

From the RATA test, two data matrices were obtained: (1) the occurrence matrix, with the number of choices of each descriptor of all subjects for each product; (2) the intensity matrix, with the sum of the intensity scores by all subjects for each attribute for each product. The occurrence matrix was analyzed with Cochran’s Q test followed by a Sheskin comparison test for multiple pairs to test whether the products differed significantly in the occurrences of their descriptors. The intensity matrix was exposed to a two-way ANOVA mixed model (random effect: subject; fixed effect: sample) to assess whether the sample significantly affects the perceived intensity of the sensory attributes. Principal Component Analysis (PAC) was applied to the intensity mean values of the significant sensory attributes to explore and visualize the data. A Penalty Analysis was conducted on the liking data and the occurrence matrix of the sensory attributes that resulted being significant from the multiple pairwise comparison tests (Sheskin) to assess which sensory attributes positively or negatively affected the liking of the six products considered together.

We set the significance level 𝛼-value to 0.05 for all the analyses. The analyses of the sensory data were conducted using the XLSTAT statistical software package version 19.04 (Addinsoft, New York, NY, USA).

#### 2.4.2. Volatile Composition Data

The analyses of the VOC data were performed by Principal Component Analysis (PCA) using the software Simca P + v.12 (Umetrics, Umeå, Sweden). The raw data were Log-transformed and then scaled to the square root of the standard deviation (Pareto scaling).

#### 2.4.3. Relationship between Sensory and VOC Data

Orthogonal partial least squares (OPLS) for regression analysis were applied to explore the relationship between the overall liking of all the participants (Y data set) and the perceived intensity of the attributes evaluated in the RATA test, for which the two-way ANOVA highlighted a significant effect of the product (X data set). The OPLS models were developed using the software Simca P + v.12 (Umetrics, Umeå, Sweden).

## 3. Results

### 3.1. Consumers’ Preferences for Gorgonzola Cheese

The mean values of the overall liking scores provided by all the subjects, males and females, for the six Gorgonzola cheese products tested are shown in Table 2. It is possible to see that all six products were accepted by consumers as the mean values obtained by the total of the subjects ranged from 63.8 to 69.8 and thus were above the ‘like slightly’ point of the LAM scale and close to the ‘like moderately’ verbal anchor [25].

The results of the two-way ANOVA model applied to the overall liking scores revealed a significant effect of both the Gorgonzola cheese styles (*p* = 0.046) and the production process (*p* = 0.011). Indeed, a higher liking mean value was observed for the sweet Gorgonzola cheese products (67.0) than for the piquant ones (65.5) and a higher liking mean value was found for the artisanal Gorgonzola cheese (67.0) than for the Gorgonzola cheese obtained by an industrial process (64.9).

The results of the two-way ANOVA model applied to the liking scores obtained from all subjects showed a significant effect of the sample (*p* < 0.0001). The sweet Gorgonzola cheese aged for 80 days (S80) was the most liked sample while the least liked products were S75 and P95. The other three cheese products (S70, P80, and P85) were associated with the intermediate liking mean values.

Females were associated with the mean values distributed in a wider range of the scale (from 60.3 to 71.2; approximately 11 points) than males (from 64.0 to 68.1; approximately 4 points), suggesting a higher discriminant ability of the females.

The *t*-test applied to the liking scores to investigate the gender effect on the liking of all six cheese products considered together did not reveal a significant difference (*p* = 0.071), even if a higher mean liking for males (67.0) compared to that of the females (65.6) was observed.

The two-way ANOVA models applied separately to the liking scores obtained by males and females did not reveal a significant effect of the sample for males (*p* = 0.084). On the contrary, a significant (*p* < 0.0001) effect of the sample was found for females, who provided a sample discrimination similar to that observed for the total of the consumers. In particular, females most liked two of the sweet Gorgonzola cheese products (S80 and S70).

The *t*-tests independently applied to the liking scores provided for each cheese to investigate the effect of the consumers’ gender showed a significance difference (*p* = 0.0001) only for the piquant Gorgonzola cheese aged for 95 days (P95), with the males liking it more than the females. No significant differences were observed between the males and females for the other five cheese products (*p* > 0.05).

### 3.2. Consumers’ Perception of the Sensory Properties of Gorgonzola Cheese

In Table 3, the occurrences of the sensory attributes selected for the Gorgonzola cheese products by consumers during the RATA test are illustrated. The occurrences varied from a minimum number of 41 (for ‘cooked vegetables’) to a maximum number of 326 (for ‘creamy’), corresponding to a minimum of 11% and a maximum of 91% of the participants, respectively. According to Cochran’s Q test results, 11 out of 13 sensory attributes were significant, including ‘salty’ and ‘sweet’ for taste, ‘ammonia’, ‘floral’, ‘moldy’, ‘nutty’, and ‘soapy’ for flavor and ‘creamy’, ‘grainy’ and ‘pungent’ for mouthfeel. Thus, the consumers perceived large differences in the sensory properties of the six evaluated Gorgonzola cheese products. Piquant-style products were associated with significantly higher occurrences of ‘salty’, ‘moldy’ and ‘pungent’ terms than the sweet-style cheese products. On the contrary, the sweet-style cheese products were more frequently associated with the attributes ‘sweet’ and ‘creamy’. Within the piquant products, a trend of an increase in the occurrences of three flavor attributes (‘ammonia’, ‘moldy’ and ‘soapy’) and two mouthfeel attributes (‘grainy’ and ‘pungent’) as the aging time of the cheese increased was observed, with cheese P95 showing significantly higher occurrences than P80. Conversely, the occurrences of ‘sweet’ tended to decrease as the aging time increased, with sample P80 showing the highest value. Similar increase/decrease trends as a function of the aging time within the sweet-style products were not observed.

The results of the two-way ANOVA mixed model applied to the intensity scores provided by the consumers for the six cheese products on gLMS during the RATA test are shown in Table 3. Generally, the intensity means of the sensory attributes were limited to the lower-half portion of the scale, with mean values ranging from 2.1 to 44.9 and approximately covering the section scale from the ‘Barely detectable’ and ‘Very strong’ intensity. A significant effect (*p* < 0.05) was found for all attributes, except for ‘cooked vegetables’. Such a non-significant attribute was excluded from further data analysis.

**Figure 1:**
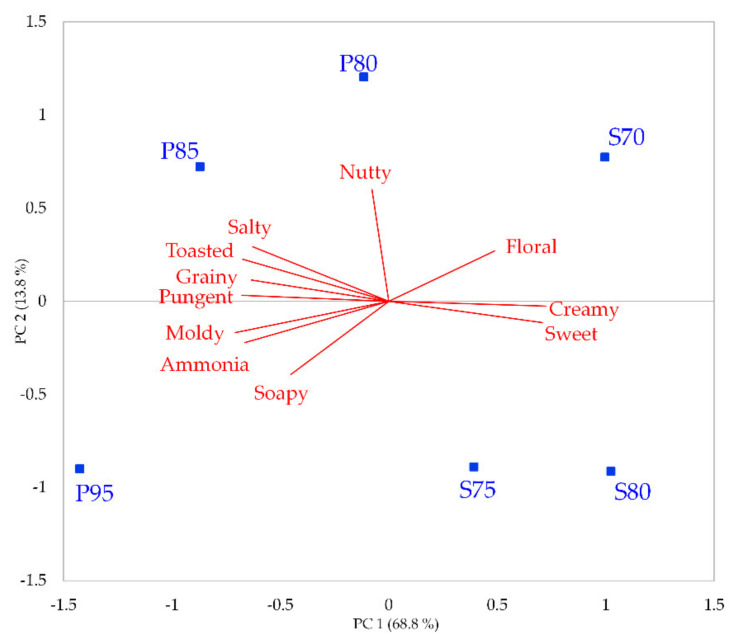
Perceptive map obtained by the PCA applied to the intensity mean values of the 11 sensory attributes evaluated with the RATA test and resulted significant from the ANOVA test (*p* < 0.05) (the only non-significant attribute was ‘cooked vegetables’).

The perceptive map obtained by the PCA, applied to the intensity mean values of the 11 sensory attributes evaluated with the RATA test that resulted being significant from the ANOVA model, is shown in Figure 1. The total variance explained for the sensory attributes based on the first two significant dimensions is 82.6%, with PC1 and PC2 accounting, respectively, for 68.8% and 13.8%. The cheese products are distributed over the whole defined sensory space, covering almost all the observed sensory variability among the Gorgonzola products. The Gorgonzola styles (sweet vs. piquant) are clearly discriminated along the first dimension. The sweet-style cheese products tend to be positively correlated with PC1 and are generally described with ‘sweet’ taste, ‘floral’ flavor, and ‘creamy’ mouthfeel. Piquant-style cheese products are negatively correlated with PC1 and described mainly as intense in ‘salty’ taste with ‘toasted’, ‘moldy’, ‘ammonia’, and ‘soapy’ flavors and with an intense ‘grainy’ and ‘pungent’ mouthfeel. In agreement with the occurrences data (Table 3), the piquant-style cheese products were significantly more intense for the attributes ‘salty’, ‘moldy’ and ‘pungent’ and less intense in terms of ‘sweet’ taste and ‘creamy’ mouthfeel than the sweet-style cheese products (Table 3). Moreover, within each cheese style, the products are distributed along the second dimension according to the increasing aging time (Figure 1). The products with lower aging times tend to be positively correlated with PC2, while the cheese products with higher aging times tend to be negatively correlated with PC2. Among the piquant products, the increasing aging time tends to increase/decrease the perceived intensity of several attributes (Table 3). For instance, ‘ammonia’, ‘moldy’, ‘soapy’, ‘grainy’, and ‘pungent’ are significantly higher in P95 than in P80, whereas the intensity of the attributes ‘sweet’ and ‘creamy’ are significantly lower in P95 than in P80. Among the sweet cheese products, the intensity of ‘moldy’ flavor is significantly higher in S80 than in S70 while the perception of the ‘toasted’ flavor is more intense in S70 than in S80.

### 3.3. Impact of the Sensory Attributes Perception on Liking

**Figure 2:**
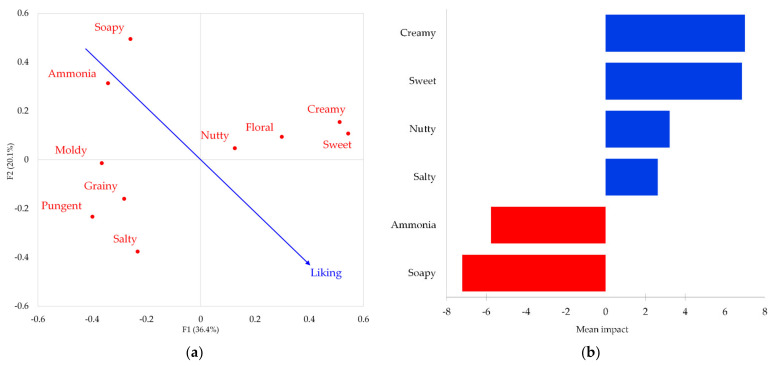
Results from the Penalty Analysis applied to the liking data and the occurrences obtained from the RATA test of the sensory attributes that resulted as being significant from the multiple pairwise comparison tests (Sheskin): (**a**) map from the Principal Coordinates Analysis (PCoA) applied to the correlation coefficient of the sensory attributes; (**b**) sensory attributes with a significant mean impact on liking (mean increases are displayed in blue while mean decreases are displayed in red).

The results from the Penalty Analysis on the liking data and the significant occurrences obtained from the RATA test of the sensory attributes are illustrated in Figure 2. The map from the Principal Coordinates Analysis (PCoA) applied to the correlation coefficient of the sensory attributes (Figure 2a) shows the relationship between the sensory attributes and the overall liking scores of the six tested Gorgonzola cheese products. The first two latent dimensions of the model explain 56.5% of the total variance, with F1 and F2 accounting, respectively, for 36.4% and 20.1%. The map displays that liking is positively associated with the RATA terms ‘sweet’, ‘creamy’, ‘floral’, ‘nutty’ and ‘salty’, and negatively related to the attributes ‘soapy’, ‘ammonia’, ‘moldy’, ‘grainy’, and ‘pungent’. Accordingly, the chart showing the attributes with a significant impact on liking (Figure 2b) identifies the ‘creamy’, ‘sweet’, ‘nutty’, and ‘salty’ attributes as ‘nice to have’ properties or drivers of liking as their perception positively impacted on liking, increasing it by 3–7 points on the used gLMS scale. On the contrary, the ‘soapy’ and ‘ammonia’ flavors are two significant attributes identified as ‘must not have’ properties or drivers of disliking as their perception negatively impacted on liking, decreasing it by 6–7 points.

### 3.4. Volatile Composition of the Gorgonzola Cheese

**Figure 3:**
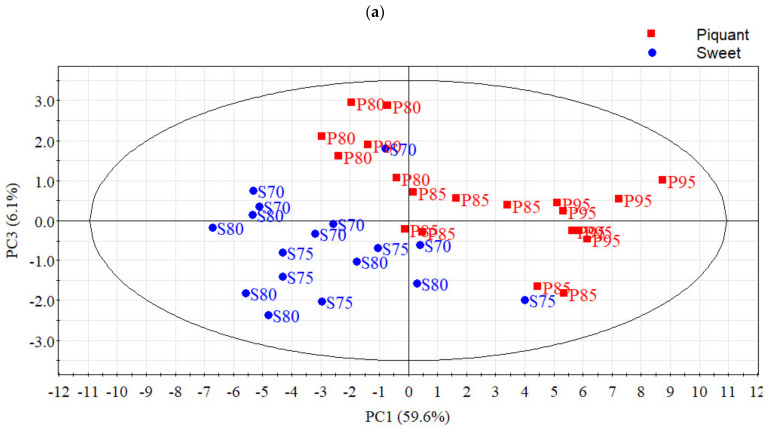

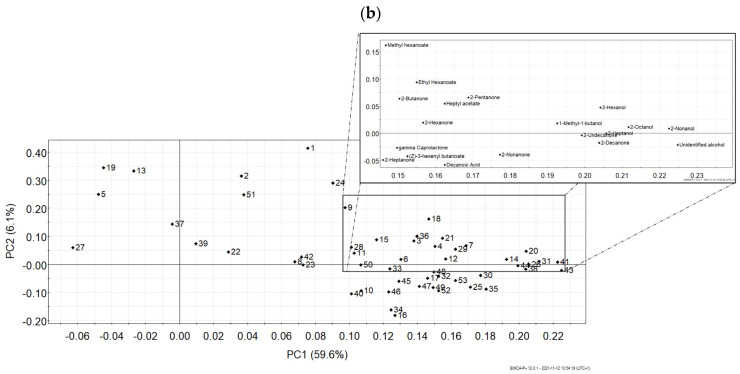
Scores (**a**) and loadings (**b**) plots of the 1st and 3rd principal components obtained from the PCA applied to VOCs data.

In the headspace of the Gorgonzola products, 53 compounds were identified (or partially identified, as in the case of an unidentified alcohol). The most represented class of molecules was alcohols with 16 compounds, followed by ketones with 13 compounds, and esters with 9 compounds; the other chemical classes were acids with 5 compounds, aromatic hydrocarbons with 4 compounds, sulfurs with 3 compounds, 1 hydrocarbon, 1 aldehyde, and 1 lactone. The detailed list of identified VOCs and their relative amounts per each of the analyzed cheese products is reported in the Supplementary Tables S1 and S2. The result of the PCA provides an overview of the cheese differences due to the VOCs, with the first three components explaining 74% of the total variance. PC1 and PC3, accounting for 65.7% of total variance, resulted in the best dimensions for clearly showing the difference between the sweet and piquant products on the score plot (Figure 3a). According to the loading plot (Figure 3b), piquant Gorgonzola cheese products are characterized by a generalized higher concentration of volatile compounds. Along the first component of the score plot, we can also observe that within the piquant variant there is an increase in volatile compounds with aging time while in the sweet variant this trend was not present. The major contribution to the VOCs differences between the two Gorgonzola styles is given by the secondary alcohols produced by the β-oxidation of free fatty acids (namely butan-2-ol, heptan-2-ol, nonan-2-ol, hexan-2-ol, octan-2-ol) and by methyl hexanoate that are more abundant in piquant Gorgonzola (Figure 3b).

### 3.5. Relationship between Volatile Composition, Sensory Attributes, and Liking

**Figure 4:**
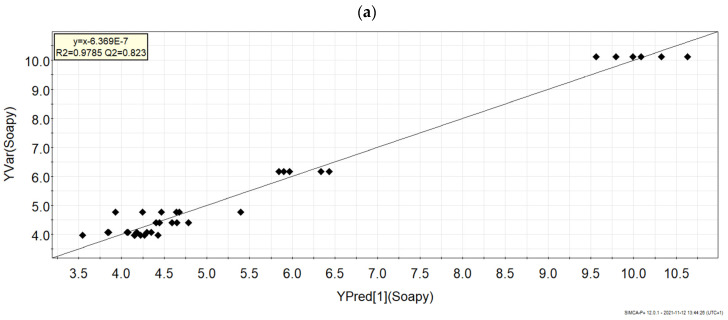

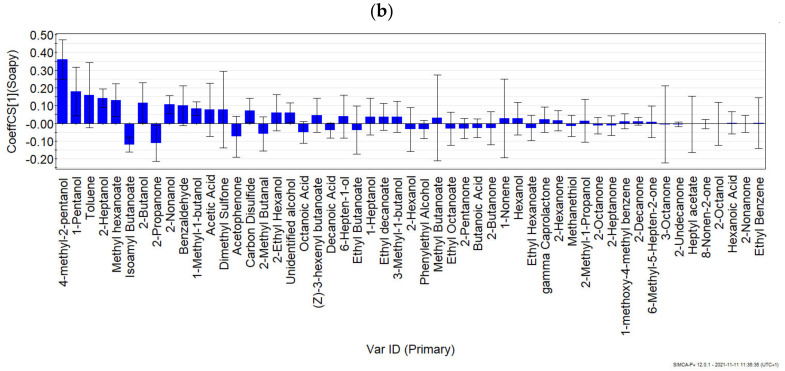
Regression model of the ‘soapy’ flavor attribute. (**a**) Plot of the observed versus predicted values of ‘soapy’ flavor perception. (**b**) Column plots of coefficients, referring to scaled and cantered data, with jack-knife uncertainty bars.

The relationship between volatile composition, sensory attributes, and liking was studied by OPLS regression models. As an example, the plot in Figure 4a displays the observed versus the predicted values of the regression model for the ‘soapy’ flavor attribute. The R^2^ = 0.979 indicates the suitability of the model also performing well after cross-validation (Q^2^ = 0.823). Once the suitability of the model was verified, the model coefficients were explored in order to identify the compounds with the highest contribution to each of the models. In Figure 4b, the coefficients for the ‘soapy’ flavor model are shown. The column plots with the jack-knife error bar not passing zero on the Y-axis indicate the coefficients significantly contributing to the model. In the case of the ‘soapy’ flavor model, 4-methyl-2-pentanol, 1-pentanol, 2-heptanol, and methyl hexanoate provide the most significant positive contribution to the model, while isoamylbutanoate and 2-propanone (acetone) give a significant negative contribution. This means that these compounds are likely to contribute to shaping the ‘soapy’ flavor in Gorgonzola. The same procedure described for the ‘soapy’ attribute was applied to all the other sensory attributes considered and liking as well. Table 4 reports a summary of the main parameters of the developed models and the first four most contributing coefficients. A more detailed table with a complete list of the coefficients is reported in Supplementary Table S3.

Together, the ‘soapy’ and ‘ammonia’ flavors attribute contribute to the Gorgonzola cheese disliking as well. The main positive contributors to the ammonia model reported in Table 4 are methyl hexanoate, 4-methyl-2-pentanol, dimethyl sulfone, and methanethiol. According to the results of the Penalty Analysis (Figure 2a), the contribution of the other measured attributes is less important and in general each of these attributes has a similar weight. As a consequence, the direction of “liking” in Gorgonzola is opposite to ‘soapy’ and ‘ammonia’. In the case of the model for liking, we found that the major contributors are the ethyl hexanoate, the (z)-3-hexenyl butanoate, the decanoic acid, and the phenylethyl alcohol.

## 4. Discussion

### 4.1. Factors Influencing Consumers’ Preferences for Gorgonzola Cheese

In this study, the consumers’ preferences and the effect of gender on liking for six Gorgonzola PDO cheese products differing in terms of style (sweet vs. piquant), aging time of the cheese wheels (70–95 days), and the production process (artisanal vs. industrial) were investigated to explore the cheese factors playing a role in the Gorgonzola acceptance.

Our study highlighted that liking was influenced by the production process, with the artisanal Gorgonzola cheese products turning out to be more appreciated than the industrial ones. This finding is in agreement with a previous work reporting that in both blind (as in our case) and expected conditions, Anger consumers gave higher liking scores for craft Maroilles cheese than for the industrial Maroilles [17]. Our result also seems to go in the direction pointed out by another study [32], which reported that industrial mass-production implies for consumers a reduction in food quality, particularly in terms of flavor. Industrial cheese products (P80 and S75) in our study, even if belonging to different styles (piquant vs. sweet) show lower differences between them and a lower concentration in volatile compounds compared to the other products. Indeed, sensory motive is the main key factor influencing consumer perception of craft foods [33]. Moreover, consumers tend to be concerned about how food is produced, preferring the artisanal production methods [34].

Consumers generally liked the sweet-style Gorgonzola cheese products more than the piquant-style ones. This result is in agreement with the production volumes of the two styles of Gorgonzola cheese every year. Indeed, out of the total volume of PDO Gorgonzola cheese produced during 2020 (over 5 million wheels) only 11.3% of the wheels belonged to the piquant style, while the most numerous of the produced wheels were of the sweet style [35], in accordance with the consumers’ preference for the sweet style. Similarly to the preference observed for the sweet Gorgonzola style, which has a more delicate taste and a less pungent flavor [6], among several types of full-fat cheese, the mild-cheese category of products received higher overall liking scores than the sharp-cheese category products [36]. Corresponding results were obtained from an affective test performed with Swiss consumers on goat and sheep cheese aimed at comparing two typical samples of each style of cheese, one with a more intense and one with a less intense animalic aroma [37]. Indeed, the cheese with weaker intensity was favored by more than 70% of participants (in the case of goat cheese) and by more than 80% (in the case of sheep cheese). On the contrary, the liking ratings expressed by consumers for several Parmigiano Reggiano PDO cheese samples aged for 24 months (with less intense sensory properties) and for 36 months (with more intense sensory properties) did not reveal a significant effect of the product [38]. This suggests that it is not possible to generalize that ‘mild’ flavor cheese is preferred to ‘strong’ flavor cheese. In fact, for instance, for Cheddar cheese, a segment of consumers with specific likes for Cheddar cheese characterized by intense flavors was opposed to a segment of consumers generally associated with young or mild Cheddar cheese flavor [39].

For Gorgonzola cheese, the patterns of preference are not linear, depending on the joint effect of the cheese style eaten and gender. Indeed, according to previous research [16], children showed a low liking for Gorgonzola cheese, when compared to other types of famous Italian cheese, with boys liking Gorgonzola significantly more than girls. In our study, we observed an analogous gender effect for the piquant Gorgonzola cheese aged for 95 days, which was significantly liked more by males than females. In addition, the present study highlighted other two gender effects on liking scores, both related to the higher ability of females to discriminate among products than males: (i) in terms of significant differences among overall liking mean values (males did not discriminate among products) and (ii) as a use of a higher portion of the evaluation liking scale (range of approximately 11 points for females and of 4 points for males on the LAM scale). To the authors’ best knowledge, no other evidence of a better capacity of females in discriminating cheese products based on liking was previously reported. In general, scarce scientific knowledge is available on the influence of gender on consumers’ affective responses to cheese, with regard or not to the flavor intensity. A study investigating the pungency perception and the liking for pasta filata cheese in consumers from different Italian regions found that females more often declared a preference for sweet cheese over pungent cheese [40]. Other authors [37] analyzed by gender their hedonic data on goat and sheep cheese but did not show any significant difference with regard to preference.

### 4.2. Consumers’ Perception of the Gorgonzola Cheese Sensory Properties

Previous research investigated how consumers described the perceived sensory properties of several PDO cheese varieties, such as Parmigiano Reggiano [38], Nostrano Valtrompia PDO Cheese [41], Swiss goat and sheep cheese [37], and Cheddar cheese [42,43], as well as some blue-veined cheese [12]. However, no specific information was previously reported about the consumers’ description of Gorgonzola cheese sensory attributes; thus, in this study, the RATA test was applied with the aim of providing a first description of the consumers’ sensory perception of this PDO cheese.

Consumers mainly associated the sweet-style Gorgonzola cheese products with the attributes ‘sweet’ and ‘creamy’, both in terms of occurrences and intensities. This result was expected since those two attributes are peculiar to this Gorgonzola cheese style, which on the market is named precisely as ‘sweet’ or ‘creamy’ [6]. Accordingly, a sweet-style Gorgonzola cheese, tested in a previous study, resulted in being sweet and slightly piquant with a creamy and soft mouthfeel [14]. The piquant-style Gorgonzola cheese products were described with the highest intensities for the attributes ‘salty’, ‘moldy’, and ‘pungent’. This is perfectly in line with the three-product high identity traits (moldy, salty, and sharp) previously identified for Gorgonzola cheese, as well as for other cow’s milk blue-veined cheese from England (Stilton), the USA, and Denmark and for a goat’s milk blue-veined cheese from France (Roquefort) [44]. Taking into consideration all the Gorgonzola cheese products tested, more specific flavor attributes were relevant to discriminate among products, such as ‘ammonia’, ‘floral’, ‘nutty’, and ‘soapy’, suggesting a good ability of the consumers in interpreting the sensory attributes and in revealing subtle flavor differences among products. The sensory attributes ‘nutty’, ‘moldy’, and ‘ammonia/ammoniated’ have been previously associated with Gorgonzola cheese [16]. Similarly, the term ‘soapy’ was previously used to describe the Gorgonzola cheese odor [6]. Moreover, the ‘soapy’ flavor has been detected in Cheddar cheese and defined as *‘A detergent-like taste. Similar to when a food is tainted with a cleansing agent’* [45,46]. In Cheddar cheese, the intensity of the ‘soapy’ flavor was affected by the milk source, with cow’s milk inducing a stronger perception than buffalo milk, while it was not influenced by either the type of starter cultures (commercially available vs. indigenous) or the ripening temperature (4 °C vs. 12 °C) [47]. Differences in terms of the ‘soapy’ flavor were observed as a function of the ripening time among several lipase-treated types of Cheddar cheese analyzed over three months but not with a clear increasing or decreasing effect [48]. In the present study, the intensity of the ‘soapy’ flavor of the piquant-style Gorgonzola cheese was affected by the aging time, with the highest aged cheese associated with a stronger intensity.

The clear distribution of the Gorgonzola cheese products on the sensory map as a function of the cheese style and aging time confirmed the potential of the application of the RATA test with consumers for sensory product-characterization purposes [26]. Our results are in agreement with previous research on Parmigiano Reggiano PDO cheese showing that the RATA test resulted in an effective rapid sensory technique to obtain consumers’ cheese discrimination as a function of the aging time [38]. The same study also pointed out that the combined collection of RATA and liking provided complementary information able to reveal the drivers of the liking/disliking of Parmigiano Reggiano cheese. Accordingly, a similar methodological approach to that used in the present study allowed investigating the drivers of the liking and disliking of Gorgonzola cheese. The Penalty Analysis applied to the liking data and the RATA responses clearly identified four sensory drivers of liking related to the (‘sweet’ and ‘salty’) taste, (‘nutty’) flavor, and (‘creamy’) mouthfeel. As it is well known that drivers of liking can vary as a function of different cheese types and clusters of consumers [12,42,49], our results are in agreement with the previous literature on cheese preferences. For instance, nutty, salty, and sweet were attributes positively correlated with the overall liking of Swiss cheese [50]. Similarly, nutty, as a typical aged flavor in cheese, was also desirable to young Chinese consumers [51]. The positive impact of sweetness and creaminess on liking confirmed the results previously observed for Gorgonzola cheese and other types of Italian PDO cheese (Mozzarella, Pecorino, Taleggio, Fontina, and Parmigiano Reggiano) evaluated by children [16]. Despite the various types of blue-veined cheese, they are mainly distinguished by their typical sensory properties and blue flavor [52]; some consumers may dislike or reject this type of cheese because of its peculiarities [12]. The literature documents several attributes related to Gorgonzola disliking, such as the appearance, saltiness, off-flavor, smell, and the disgusting and rotten taste [18]. In the present study, none of the attributes related to taste and mouthfeel significantly affected the consumers’ affective response. On the contrary, the negative impact on liking was due mainly to two significant flavor attributes (‘soapy’ and ‘ammonia’). This result is in agreement with previous studies reporting that the sensory characteristic of ‘soapy’’ would not usually be associated with an acceptable Cheddar product [45]. Moreover, some research defined the ‘soapy’ flavor as an off-flavor of cheese [48,53], indicating a clear negative characteristic.

### 4.3. VOC and Relationship with Sensory Perception

The VOCs found in our study have already been reported previously as important odor-impact molecules for Gorgonzola cheese [6]. Here, we also compared the two versions of Gorgonzola cheese, sweet vs. piquant. The compounds characterizing piquant Gorgonzola are derived by the oxidation of fatty acids. Such compounds are well documented in the literature, among them heptan-2-ol was identified as a key odorant of Gorgonzola [54].

In the present study, we found an association between single sensory descriptors and volatile compounds. For all the sensory attributes, except for ‘cooked vegetables’ and ‘nutty’, it was possible to build a reliable regression model based on the measured VOCs. The same volatile compound may contribute to different sensory attributes with a different weight. We know that odors and flavors are the results of a combination of different volatile compounds strongly influenced by the relative amount of the different components and their interactions [55]. On the other hand, the association between VOCs and sensory descriptors does not always imply causation.

The ‘soapy’ and ‘ammonia’ flavors are the major drivers of disliking in Gorgonzola cheese and the four main VOCs associated with ‘ammonia’ are 4-methyl-2-pentanol, methyl hexanoate, dimethyl sulfone, and methanethiol. Three of them (4-methyl-2-pentanol, dimethyl sulfone and methanethiol) originate from amino acid metabolism [56,57] that also brings to liberation the ammonia or ammines that are not detected by our measurements and are most likely responsible of the ‘ammonia’ flavor perception. The chromatographic conditions we adopted in our study did not allow us to detect the ammonia molecule directly but its presence is suggested by the occurrence of the other amino acid catabolites highlighted by the regression model (4-methyl-2-pentanol, dimethyl sulfone, and methanethiol). The major compounds related to the ‘soapy’ flavor are 4-methyl-2-pentanol, 1-pentanol, 2-heptanol, and methyl hexanoate. In the literature, the ‘soapy’ flavor in Gouda cheese has been associated with the presence of long-chain free fatty acids (mainly C14-C18) induced by the mold lipolysis [58]. The synergy among the volatile compounds and the free fatty acids may explain the observed differences in the ‘soapy’ flavor intensity among the investigated products.

As the ‘soapy’ and ‘ammonia’ flavors are negatively correlated with liking, we expect that the major contributors or other compounds originating from the same catabolic process models would have a negative impact on the model developed for ‘liking’. As expected, 4-methyl-2-pentanol (a major contributor for both ‘soapy’ and ‘ammonia’) has the highest negative impact on the ‘liking’ model, followed by 3-methyl-1-butanol and 2-methyl-1-propanol, two other compounds derived from amino acid catabolism [56,57]. Conversely, the major positive contributors to the ‘liking’ model are ethyl hexanoate, (z)-3-hexenyl butanoate, decanoic acid, and phenylethyl alcohol. It is interesting to note that the two esters and the phenylethyl alcohol are associated with fruity (esters) or rose (phenylethyl alcohol) odor descriptors, at least when present in pure solution [59].

## 5. Conclusions

In conclusion, this study provided new knowledge on the influence of the cheese style, aging time, and production process on the consumers’ sensory perception and the volatile composition of Gorgonzola PDO cheese. The identification of the positive and negative sensory drivers of acceptance extends the understanding of the consumers’ affective response to Gorgonzola cheese. Moreover, the combined sensory and instrumental approach used in this research allowed identifying the main VOCs potentially contributing to sensory descriptors and liking. Overall, the obtained results on Gorgonzola cheese opened up a better understanding of consumers’ preferences for blue-veined cheese in general. In future, it would be interesting to investigate the individual differences in blue-veined cheese and sensory perception, particularly regarding the attributes responsible for a decrease in liking, such as the ‘soapy’ and ‘ammonia’ flavors.

## Figures and Tables

**Table 1:** Characteristics and codes of the Gorgonzola cheese products.

Code	Style	Aging Time (Days)	Production Process
S80	Sweet	80	Artisanal
S75	Sweet	75	Industrial
S70	Sweet	70	Artisanal
P95	Piquant	95	Artisanal
P85	Piquant	85	Artisanal
P80	Piquant	80	Industrial

**Table 2:** Consumers’ overall liking mean values obtained from all subjects (*n* = 358), males (*n* = 163) and females (*n* = 195) for six Gorgonzola cheese products.

Group	S80	S75	S70	P95	P85	P80	*p*-Value (ANOVA)
Total	69.8 ^a^	63.8 ^c^	67.5 ^ab^	63.9 ^c^	66.7 ^abc^	65.9 ^bc^	<0.0001
Males	68.1	64	67.2	68.1	67.3	67.5	0.084
Females	71.2 ^a^	63.7 ^bc^	67.7 ^ab^	60.3 ^c^	66.3 ^bc^	64.6 ^bc^	<0.0001
*p*-value (*t*-test)	0.0866 ^ns^	0.8868 ^ns^	0.8018 ^ns^	0.0001 ***	0.5747 ^ns^	0.156 ^ns^	

Different letters within columns indicate significant different mean values (Tukey’s HSD test, *p* < 0.05). Results from the *t*-test to check the gender effect on cheese liking: ns indicates not a significant difference (*p* > 0.05); *** indicates the high significant difference (*p* < 0.001).

**Table 3:** Sensory attributes selected for the Gorgonzola cheese products by consumers during the RATA test. For each attribute was reported: the Cochran’s Q test *p*-value, the occurrences (OC, percentage in brackets), the significance values from the multiple pairwise comparison tests (Sheskin, S), the means of the intensity scores (M), and the *p*-value from the two-way ANOVA model.

Attributes	*p*-Value	S80					S75					S70					P95					P85					P80					*p*-Value
	Cochran’s Q Test	OC	S		M		OC	S		M		OC	S		M		OC	S		M		OC	S		M		OC	S		M		ANOVA
*Taste*																																
**Salty**	**<0.0001**	**182 (51)**	**0.508**	**^a^**	**10.8**	**^c^**	**181 (51)**	**0.506**	**^a^**	**10.4**	**^c^**	**167 (47)**	**0.466**	**^a^**	**8.9**	**^c^**	**257 (72)**	**0.718**	**^b^**	**24.4**	**^b^**	**293 (82)**	**0.818**	**^c^**	**31.3**	**^a^**	**268 (75)**	**0.749**	**^bc^**	**25.5**	**^b^**	**<0.0001**
**Sweet**	**<0.0001**	**207 (58)**	**0.578**	**^c^**	**17.3**	**^a^**	**180 (50)**	**0.503**	**^c^**	**11.9**	**^b^**	**210 (59)**	**0.587**	**^c^**	**18.1**	**^a^**	**63 (18)**	**0.176**	**^a^**	**2.6**	**^d^**	**81 (23)**	**0.226**	**^a^**	**3.2**	**^d^**	**122 (34)**	**0.341**	**^b^**	**5.9**	**^c^**	**<0.0001**
*Flavor*																																
**Ammonia**	**<0.0001**	**106 (30)**	**0.296**	**^a^**	**4.9**	**^c^**	**114 (32)**	**0.318**	**^a^**	**7.0**	**^b^**	**94 (26)**	**0.263**	**^a^**	**4.8**	**^c^**	**153 (43)**	**0.427**	**^b^**	**12.1**	**^a^**	**111 (31)**	**0.310**	**^a^**	**7.3**	**^b^**	**105 (29)**	**0.293**	**^a^**	**7.1**	**^b^**	**<0.0001**
Cooked vegetables	0.477	54 (15)	0.151	^a^	2.9	^a^	41 (11)	0.115	^a^	2.1	^a^	51 (14)	0.142	^a^	2.3	^a^	49 (14)	0.137	^a^	3.2	^a^	44 (12)	0.123	^a^	2.1	^a^	53 (15)	0.148	^a^	3.0	^a^	0.169
**Floral**	**0.011**	**77 (22)**	**0.215**	**^ab^**	**5.0**	**^ab^**	**63 (18)**	**0.176**	**^ab^**	**3.6**	**^bc^**	**87 (24)**	**0.243**	**^b^**	**5.5**	**^a^**	**65 (18)**	**0.182**	**^ab^**	**3.6**	**^bc^**	**55 (15)**	**0.154**	**^a^**	**3.0**	**^c^**	**69 (19)**	**0.193**	**^ab^**	**5.4**	**^a^**	**0.002**
**Moldy**	**<0.0001**	**155 (43)**	**0.433**	**^ab^**	**9.2**	**^de^**	**177 (49)**	**0.494**	**^b^**	**11.2**	**^cd^**	**135 (38)**	**0.377**	**^a^**	**6.8**	**^e^**	**253 (71)**	**0.707**	**^c^**	**28.1**	**^a^**	**224 (63)**	**0.626**	**^c^**	**19.1**	**^b^**	**170 (47)**	**0.475**	**^b^**	**12.9**	**^c^**	**<0.0001**
**Nutty**	**0.003**	**66 (18)**	**0.184**	**^a^**	**3.4**	**^c^**	**75 (21)**	**0.209**	**^ab^**	**4.4**	**^bc^**	**102 (28)**	**0.285**	**^b^**	**6.1**	**^a^**	**77 (22)**	**0.215**	**^ab^**	**4.8**	**^abc^**	**80 (22)**	**0.223**	**^ab^**	**5.0**	**^abc^**	**91 (25)**	**0.254**	**^ab^**	**5.7**	**^ab^**	**0.018**
**Soapy**	**<0.0001**	**73 (20)**	**0.204**	**^a^**	**4.0**	**^c^**	**100 (28)**	**0.279**	**^ab^**	**6.2**	**^b^**	**82 (23)**	**0.229**	**^a^**	**4.8**	**^bc^**	**125 (35)**	**0.349**	**^b^**	**10.1**	**^a^**	**71 (20)**	**0.198**	**^a^**	**4.1**	**^c^**	**78 (22)**	**0.218**	**^a^**	**4.4**	**^bc^**	**<0.0001**
Toasted	0.261	60 (17)	0.168	^a^	**2.8**	^**c**^	60 (17)	0.168	**^a^**	**3.9**	^**abc**^	63 (18)	0.176	^a^	**3.5**	^**bc**^	69 (19)	0.193	^a^	**4.8**	^**ab**^	79 (22)	0.221	^a^	**5.1**	^a^	64 (18)	0.179	^a^	**4.3**	^**ab**^	**0.020**
*Mouthfeel*																																
**Creamy**	**<0.0001**	**324 (91)**	**0.905**	**^d^**	**42.1**	**^b^**	**287 (80)**	**0.802**	**^c^**	**28.6**	**^c^**	**326 (91)**	**0.911**	**^d^**	**44.9**	**^a^**	**138 (39)**	**0.385**	**^a^**	**6.8**	**^e^**	**161 (45)**	**0.450**	**^a^**	**9.2**	**^e^**	**246 (69)**	**0.687**	**^b^**	**21.5**	**^d^**	**<0.0001**
**Grainy**	**<0.0001**	**88 (25)**	**0.246**	**^a^**	**4.7**	**^bc^**	**68 (19)**	**0.190**	**^a^**	**3.5**	**^c^**	**99 (28)**	**0.277**	**^a^**	**5.7**	**^b^**	**164 (46)**	**0.458**	**^b^**	**10.3**	**^a^**	**158 (44)**	**0.441**	**^b^**	**10.6**	**^a^**	**92 (26)**	**0.257**	**^a^**	**5.8**	**^b^**	**<0.0001**
**Pungent**	**<0.0001**	**145 (41)**	**0.405**	**^a^**	**8.3**	**^d^**	**142 (40)**	**0.397**	**^a^**	**8.0**	**^d^**	**120 (34)**	**0.335**	**^a^**	**5.9**	**^d^**	**259 (72)**	**0.723**	**^c^**	**27.9**	**^a^**	**204 (57)**	**0.570**	**^b^**	**17.4**	**^c^**	**221 (62)**	**0.617**	**^b^**	**20.3**	**^b^**	**<0.0001**

The attributes that resulted as significant from the Cochran’s Q test and the two-way ANOVA model are reported in bold. In the rows, values identified by different lowercase superscript letters indicate significant differences from the Sheskin’s multiple pairwise comparison tests. In rows, the values identified by different uppercase superscript letters indicate significantly different mean values (Tukey’s HSD Test, *p* < 0.05).

**Table 4:** Main parameters of the regression models of the sensory attributes and first four most contributing coefficients.

Attributes.	Equations	R^2^	Q^2^	*p* _(CV−ANOVA)_	Variable Coefficient (Significant)
*Taste*					
Salty	y = x + 8.818E ^−7^	0.448	0.329	0.001	isoamyl butanoate; 2-propanone; (z)-3-hexenyl butanoate; 2-pentanone
Sweet	y = x − 1.394E ^−6^	0.963	0.835	<0.001	2-butanol; 2-methyl butanal; 1-pentanol; ethyl octanoate
*Flavor*					
Ammonia	y = x − 1.613E ^−7^	0.976	0.912	<0.001	4-methyl-2-pentanol; methyl hexanoate; dimethyl sulfone; methanethiol
Cooked vegetables	n.s.			n.s.	
Floral	y = x − 9.763E ^−8^	0.914	0.619	0.002	2-butanol; 2-methyl butanal; 1-pentanol; ethyl octanoate
Moldy	y = x − 2.704E ^−7^	0.974	0.867	<0.001	(z)-3-hexenyl butanoate; dimethyl sulfone; methyl hexanoate; methanethiol
Nutty	n.s.			n.s.	
Soapy	y = x − 6.369E ^−7^	0.979	0.823	<0.001	4-methyl-2-pentanol; 1-pentanol; 2-heptanol; methyl hexanoate
Toasted	y = x + 1.89E ^−7^	0.920	0.749	<0.001	3-octanone; ethyl benzene; 2-ethyl hexanol; 1-methyl-1-butanol
*Mouthfeel*					
Creamy	y = 1*x − 8.436E ^−7^	0.960	0.833	<0.001	2-methyl butanal; 1-pentanol; 2-butanol; 1-methoxy-4-methyl benzene
Grainy	y = x − 1.961E ^−7^	0.601	0.517	<0.001	(z)-3-hexenyl butanoate; 1-methyl-1-butanol; ethyl hexanoate; 2-octanol
Pungent	y = x − 1.544E ^−7^	0.985	0.879	<0.001	methyl hexanoate; dimethyl sulfone; methanethiol; isoamyl butanoate
*Liking*	y = x − 1.478E ^−5^	0.933	0.628	0.008	ethyl hexanoate; (z)-3-hexenyl butanoate; decanoic acid; phenylethyl alcohol

## Data Availability

The data presented in this study are available on request from the corresponding author.

## Supplementary Materials

The following are available online at https://www.mdpi.com/article/10.3390/foods10112791/s1, Table S1: VOCs identified and their relative amounts (μg equivalent of I.S.) in each Sweet style Gorgonzola PDO cheese, Table S2: VOCs identified and their relative amounts (μg equivalent of I.S.) in each Piquant style Gorgonzola PDO cheese, Table S3: Regression models from orthogonal partial least squares applied to investigate the relationship between volatile composition and liking and sensory attributes.
