# Supplementary material for: Relationship between Sensory Attributes, (Dis) Liking and Volatile Organic Composition of Gorgonzola PDO Cheese
Source: Foods. 2021 Nov 12;10(11):2791. doi: 10.3390/foods10112791 (PMC8621326; doi:10.3390/foods10112791)
Supplement: Supplementary file 1 [file foods-10-02791-s001.zip › Supplementary Table 1_rev.pdf]

Supplementary Table S1. VOCs identified and their relative amounts (μg equivalent of I.S.) in each Sweet style Gorgonzola PDO cheese.

N.	Compound	LRIc	LRI(NIST)	S80				S70				S75			
				Mean	Min	Max	CV	Mean	Min	Max	CV	Mean	Min	Max	CV
1	Methanethiol	792	692	1	0	2	140	4	0	9	137	0	0	0	0
2	Carbon disulfide	797	735	108	0	322	141	209	0	802	166	34	0	135	200
3	2-Propanone	821	819	51	28	105	63	100	51	175	53	249	53	744	133
4	2-Butanone	908	907	10	5	21	69	18	9	34	57	28	5	86	140
5	2-Methyl butanal	922	914	89	61	122	34	240	124	442	58	83	44	116	38
6	1-Nonene	937	950	33	0	77	115	49	20	104	67	85	21	171	84
7	2-Pentanone	985	981	1416	295	3562	100	4012	864	9434	97	8618	287	28665	157
8	Methyl butanoate	997	982	0	0	0	0	0	0	0	0	0	0	0	0
9	2-Butanol	1029	1025	4	0	7	72	7	4	10	32	0	0	0	0
10	Ethyl butanoate	1045	1035	98	23	226	96	111	39	268	91	77	21	138	69
11	Toluene	1047	1042	9	6	14	31	13	8	22	43	11	8	13	25
12	2-Hexanone	1091	1083	162	46	389	93	302	92	678	81	589	69	1815	140
13	2-Methyl-1-propanol	1103	1092	24	17	30	20	75	54	102	25	58	36	100	49
14	1-Methyl-1-butanol	1129	1119	81	44	160	62	127	44	216	57	318	54	934	131
15	Ethyl benzene	1135	1129	1	0	2	141	1	0	3	224	2	0	8	200
16	4-methyl-2-pentanol	1176	1168	1	0	4	151	2	0	8	224	23	12	42	59
17	2-Heptanone	1192	1182	16701	2869	38711	88	23536	6645	58517	90	44589	8160	125630	124
18	Methyl hexanoate	1197	1184	0	0	0	0	0	0	0	0	0	0	0	0
19	3-Methyl-1-butanol	1217	1209	1498	803	1860	27	3816	3306	4879	18	2905	1439	4895	51
20	2-Hexanol	1231	1220	4	0	7	82	8	0	17	99	15	0	56	179
21	Ethyl hexanoate	1244	1233	119	9	312	109	179	50	377	83	66	0	174	119
22	1-Pentanol	1261	1250	76	33	160	65	81	67	100	15	140	72	234	50
23	3-Octanone	1263	1253	9	0	19	106	22	0	43	85	46	30	83	54
24	Isoamyl butanoate	1275	1259	89	29	236	95	159	78	262	52	84	26	155	65
25	2-Octanone	1294	1287	458	50	1264	107	511	95	1458	108	2200	245	7137	151
26	2-Heptanol	1330	1320	235	55	524	87	546	105	1177	86	1638	139	4925	135
27	6-Methyl-5-hepten-2-one	1348	1338	16	13	21	19	30	19	37	30	31	18	46	42
28	Hexanol	1366	1355	2	0	4	137	3	0	11	146	0	0	0	0
29	Heptyl acetate	1385	1377	4	0	17	190	3	0	9	138	13	0	41	145
30	2-Nonanone	1399	1390	17553	1820	47902	107	24547	3786	70731	111	70989	8256	231033	151
31	2-Octanol	1430	1412	5	0	18	147	4	0	18	183	28	0	113	200
32	(Z)-3-hexenyl butanoate	1439	1454	5	0	17	149	3	0	17	224	0	0	0	0
33	Ethyl octanoate	1445	1435	127	58	317	85	139	78	247	54	113	31	205	70
34	1-methoxy-4-methyl benzene	1449	1434	192	83	420	70	157	89	284	52	286	128	699	97
35	8-Nonen-2-one	1454	1484	2593	181	8472	132	2904	365	9471	130	15461	1014	55321	172
36	1-Heptanol	1468	1453	41	8	130	126	34	14	61	55	72	22	136	66
37	2-Ethyl hexanol	1496	1491	0	0	0	0	1	0	6	224	8	0	14	73
38	2-Decanone	1503	1494	102	11	360	145	123	17	368	119	671	23	2433	175
39	Acetic acid	1510	1449	3	0	16	224	24	0	48	79	34	0	136	200
40	6-Hepten-1-ol	1524	-	5	0	21	165	0	0	0	0	19	7	42	80
41	2-Nonanol	1531	1521	95	11	243	104	183	27	333	79	940	37	3347	171
42	Benzaldehyde	1533	1520	1	0	4	112	3	0	6	71	0	0	0	0
43	Unidentified alcohol	1583	***	15	0	49	144	30	0	74	101	234	0	880	185
44	2-Undecanone	1607	1598	869	146	2994	140	1393	204	3937	112	6489	188	23589	176
45	Ethyl decanoate	1648	1638	25	12	54	69	28	12	55	62	44	16	95	82
46	Acetophenone	1659	1647	1	0	4	141	1	0	3	224	3	0	10	151
47	Butanoic acid	1666	1625	285	49	707	93	314	50	949	116	657	119	1539	102
48	γ-caprolactone	1707	1694	22	3	69	120	33	6	106	126	93	12	287	141
49	Hexanoic acid	1882	1846	306	54	770	94	275	80	643	85	873	109	2286	115
50	Dimethyl sulfone	1908	1903	5	2	7	53	3	1	6	49	10	5	14	47
51	Phenylethyl alcohol	1921	1906	72	21	147	82	93	58	146	40	110	23	184	62
52	Octanoic acid	2132	2060	111	22	264	87	103	26	269	99	177	27	493	122
53	Decanoic acid	2301	2276	54	11	148	100	53	11	138	98	76	13	223	130

LRIc: calculated linear retention index; LRI(NIST): linear retention index from NIST database; CV: coefficient of variation (%).
