# Supplementary material for: Relationship between Sensory Attributes, (Dis) Liking and Volatile Organic Composition of Gorgonzola PDO Cheese
Source: Foods. 2021 Nov 12;10(11):2791. doi: 10.3390/foods10112791 (PMC8621326; doi:10.3390/foods10112791)
Supplement: Supplementary file 1 [file foods-10-02791-s001.zip › Supplementary Table 2_rev.pdf]

Supplementary Table S2. VOCs identified and their relative amounts (μg equivalent of I.S.) in each Piquant style Gorgonzola PDO cheese.

N.	Compound	LRIc	$LRI_{(NIST)}$	P95				P85				P80			
				Mean	Min	Max	CV	Mean	Min	Max	CV	Mean	Min	Max	CV
1	Methanethiol	792	692	20	14	31	31	2	0	6	155	14	8	23	42
2	Carbon disulfide	797	735	758	0	2348	143	705	0	2893	161	379	0	1168	143
3	2-Propanone	821	819	505	189	1343	84	233	152	451	49	185	117	243	27
4	2-Butanone	908	907	111	56	174	41	28	14	49	44	23	12	38	45
5	2-Methyl butanal	922	914	55	23	124	69	130	53	273	66	200	85	356	56
6	1-Nonene	937	950	293	194	367	22	143	0	506	158	74	52	102	31
7	2-Pentanone	985	981	18186	8271	27774	42	12031	6330	25784	60	3747	1193	5189	49
8	Methyl butanoate	997	982	205	0	422	86	0	0	0	0	0	0	0	0
9	2-Butanol	1029	1025	41	18	66	46	3	0	18	245	7	0	12	67
10	Ethyl butanoate	1045	1035	340	62	775	101	397	16	1056	98	71	25	154	73
11	Toluene	1047	1042	41	27	65	32	15	10	21	30	12	9	17	27
12	2-Hexanone	1091	1083	1374	765	2114	33	792	506	1430	44	309	129	499	49
13	2-Methyl-1-propanol	1103	1092	34	27	43	17	46	25	79	47	185	126	225	21
14	1-Methyl-1-butanol	1129	1119	4618	2103	7678	47	686	300	1768	82	151	89	208	32
15	Ethyl benzene	1135	1129	19	12	29	36	9	6	11	28	8	0	22	116
16	4-methyl-2-pentanol	1176	1168	234	156	301	25	1	0	6	245	0	0	0	0
17	2-Heptanone	1192	1182	86063	5874	11909	29	80633	5285	12477	41	21764	1021	32671	43
18	Methyl hexanoate	1197	1184	1805	956	3265	51	24	0	143	245	93	68	115	22
19	3-Methyl-1-butanol	1217	1209	1452	1211	1944	18	3114	1785	4828	42	10199	6935	12368	22
20	2-Hexanol	1231	1220	423	217	576	33	24	10	58	72	7	0	13	64
21	Ethyl hexanoate	1244	1233	1140	300	2187	66	1304	30	3903	107	171	57	297	62
22	1-Pentanol	1261	1250	157	98	232	31	51	36	85	34	108	68	172	41
23	3-Octanone	1263	1253	42	24	62	40	139	56	201	40	53	26	76	47
24	Isoamyl butanoate	1275	1259	340	234	452	24	511	193	773	42	675	422	913	31
25	2-Octanone	1294	1287	4155	2487	8884	59	3219	1544	6507	64	549	298	968	48
26	2-Heptanol	1330	1320	26513	1461	37321	36	1648	544	4762	96	368	209	616	41
27	6-Methyl-5-hepten-2-one	1348	1338	10	0	27	120	21	0	41	64	32	25	37	15
28	Hexanol	1366	1355	31	25	36	16	5	0	17	159	5	0	10	95
29	Heptyl acetate	1385	1377	87	41	196	68	11	0	33	108	7	6	12	35
30	2-Nonanone	1399	1390	19227	1196	38515	55	12750	5515	26774	64	27083	1257	54608	62
31	2-Octanol	1430	1412	592	358	814	35	30	15	73	77	9	6	13	31
32	(Z)-3-hexenyl butanoate	1439	1454	81	34	177	68	32	14	59	65	2	0	11	224
33	Ethyl octanoate	1445	1435	524	240	754	40	407	55	1419	126	138	65	190	37
34	1-methoxy-4-methyl	1449	1434	729	451	1335	47	279	150	600	63	127	60	235	55
35	8-Nonen-2-one	1454	1484	24298	1011	60304	77	16939	6251	38253	73	2629	1239	5525	67
36	1-Heptanol	1468	1453	291	157	554	49	101	33	196	63	104	70	129	26
37	2-Ethyl hexanol	1496	1491	52	0	311	245	6	0	22	159	13	0	28	80
38	2-Decanone	1503	1494	2573	1362	6114	74	749	262	1768	79	152	57	339	75
39	Acetic acid	1510	1449	51	0	193	163	15	0	90	245	7	0	34	224
40	6-Hepten-1-ol	1524	-	58	0	159	94	11	0	38	140	6	0	16	137
41	2-Nonanol	1531	1521	16608	1013	22814	31	707	227	1661	84	168	108	234	30
42	Benzaldehyde	1533	1520	19	0	58	155	7	0	18	98	1	0	3	224
43	Unidentified alcohol	1583	-	1886	982	2849	40	125	28	315	89	18	12	24	29
44	2-Undecanone	1607	1598	22591	1096	47599	63	6314	2149	11543	68	1582	534	3503	76
45	Ethyl decanoate	1648	1638	129	49	218	47	63	14	185	101	27	14	41	43
46	Acetophenone	1659	1647	15	0	33	75	4	0	15	160	0	0	0	0
47	Butanoic acid	1666	1625	1051	667	1658	38	1610	419	3498	68	264	170	366	32
48	γ-caprolactone	1707	1694	106	45	268	78	164	74	394	71	31	13	64	64
49	Hexanoic acid	1882	1846	1484	1022	2210	38	1310	440	2607	71	314	203	433	29
50	Dimethyl sulfone	1908	1903	18	9	31	39	10	5	18	46	7	5	9	28
51	Phenylethyl alcohol	1921	1906	89	25	137	51	354	71	959	96	149	74	243	52
52	Octanoic acid	2132	2060	573	333	831	33	349	145	651	68	96	58	142	32
53	Decanoic acid	2301	2276	358	188	601	49	154	66	350	70	44	28	68	34

LRIc: calculated linear retention index; LRI(NIST): linear retention index from NIST database; CV: coefficient of variation (%).
