# Supplementary material for: Relationship between Sensory Attributes, (Dis) Liking and Volatile Organic Composition of Gorgonzola PDO Cheese
Source: Foods. 2021 Nov 12;10(11):2791. doi: 10.3390/foods10112791 (PMC8621326; doi:10.3390/foods10112791)
Supplement: Supplementary file 1 [file foods-10-02791-s001.zip › Supplementary Table 3.pdf]

Supplementary Table S3. Regression models from orthogonal partial least squares applied to investigate the relationship between volatile composition and liking and sensory attributes.

	Liking and Taste attributes					
	Liking		Sweet		Salty	
Equations	y=1*x-1.478e-005		y=1*x-1.394e-006		y=1*x+8.818e-007	
R2		0.933		0.963		0.448
Q2		0.628		0.835		0.329
p (CV-ANOVA)		0.008		<<0.001		0.001
V1	Ethyl Hexanoate	0.154	1-Pentanol	0.148	Isoamyl Butanoate	0.029
V2	(Z)-3-hexenyl butanoate	0.116	2-Methyl Butanal	0.129	2-Propanone	0.025
V3	Decanoic Acid	0.102	2-Butanol	0.117	(Z)-3-hexenyl butanoate	0.024
V4	Phenylethyl Alcohol	0.074	1-methoxy-4-methyl benzene	0.114	2-Pentanone	0.024
V5	1-Methyl-1-butanol	-0.084	Hexanol	-0.039	2-Nonanone	0.024
V6	Ethyl decanoate	-0.095	2-Octanone	-0.043	2-Decanone	0.023
V7	2-Nonanol	-0.105	2-Hexanol	-0.046	1-Methyl-1-butanol	0.023
V8	Benzaldehyde	-0.119	3-Methyl-1-butanol	-0.062	2-Octanone	0.023
V9	1-Pentanol	-0.122	1-Methyl-1-butanol	-0.070	2-Hexanone	0.023
V10	Ethyl Benzene	-0.141	Butanoic Acid	-0.071	2-Undecanone	0.023
V11	2-Heptanol	-0.179	2-Ethyl Hexanol	-0.119	2-Octanol	0.023
V12	3-Methyl-1-butanol	-0.181	Methanthiol	-0.138	Ethyl Benzene	0.023
V13	2-Methyl-1-Propanol	-0.227	Isoamyl Butanoate	-0.148	2-Heptanone	0.023
V14	4-methyl-2-pentanol	-0.423	Methyl hexanoate	-0.155	Ethyl Hexanoate	0.022
V15			3-Octanone	-0.211	1-Heptanol	0.022
V16					8-Nonen-2-one	0.022
V17					Hexanoic Acid	0.021
V18					2-Butanone	0.021
V19					Octanoic Acid	0.021
V20					Decanoic Acid	0.021
V21					Dimethyl Sulfone	0.020
V22					2-Hexanol	0.020
V23					2-Nonanol	0.019
V24					Unidentidied alcohol*	0.019
V25					2-Heptanol	0.018
V26					Methyl hexanoate	0.018
V27					Ethyl Octanoate	0.018
V28					Toluene	0.017
V29					Heptyl acetate	0.017
V30					Ethyl decanoate	0.015
V31					Hexanol	0.012

	Flavour attributes									
	Ammonia		Floral		Mouldy		Soapy		Toasted	
Equations	y=1*x-1.613e-007		y=1*x-9.763e-008		y=1*x-2.704e-007		y=1*x-6.369e-007		y=1*x+1.89e-007	
R2		0.976		0.914		0.974		0.979		0.920
Q2		0.912		0.619		0.867		0.823		0.749
p (CV-ANOVA)		<<0.001		0.002		<<0.001		<<0.001		<<0.001
V1	4-methyl-2-pentanol	0.154	2-Butanol	0.218	(Z)-3-hexenyl butanoate	0.189	4-methyl-2-pentanol	0.359	3-Octanone	0.254
V2	Methyl hexanoate	0.153	2-Methyl Butanal	0.181	Dimethyl Sulfone	0.189	1-Pentanol	0.178	Ethyl Benzene	0.157
V3	Dimethyl Sulfone	0.139	1-Pentanol	0.142	Methyl hexanoate	0.157	2-Heptanol	0.143	2-Ethyl Hexanol	0.145
V4	Methanthiol	0.096	Ethyl Octanoate	0.082	Methanthiol	0.121	Methyl hexanoate	0.130	1-Methyl-1-butanol	0.077
V5	Methyl Butanoate	0.085	2-Nonanol	-0.033	Methyl Butanoate	0.117	2-Butanol	0.116	Methyl hexanoate	0.058
V6	3-Octanone	0.079	2-Hexanol	-0.066	3-Octanone	0.095	2-Nonanol	0.106	2-Octanone	0.045
V7	1-Methyl-1-butanol	0.075	2-Octanone	-0.084	1-Methyl-1-butanol	0.093	1-Methyl-1-butanol	0.083	2-Nonanone	0.038
V8	2-Heptanol	0.074	gamma Caprolactone	-0.084	Butanoic Acid	0.054	Carbon Disulfide	0.073	Acetophenone	-0.072
V9	2-Nonanol	0.069	2-Heptanol	-0.097	2-Heptanol	0.050	Unidentidied alcohol*	0.060	2-Methyl Butanal	-0.073
V10	2-Hexanol	0.052	Butanoic Acid	-0.111	2-Nonanol	0.047	2-Propanone	-0.113	2-Butanol	-0.129
V11	Hexanol	0.051	1-Methyl-1-butanol	-0.128	1-methoxy-4-methyl benzene	-0.085	Isoamyl Butanoate	-0.121	1-Pentanol	-0.133
V12	6-Hepten-1-ol	0.048	Phenylethyl Alcohol	-0.148						
V13	Heptyl acetate	0.036								
V14	2-Decanone	0.018								
V15	Octanoic Acid	-0.022								
V16	Ethyl Octanoate	-0.040								
V17	Ethyl Butanoate	-0.069								
V18	2-Methyl Butanal	-0.087								

	Mouthfeel attributes					
	Creamy		Grainy		Pungent	
Equations	y=1*x-8.436e-007		y=1*x-1.961e-007		y=1*x-1.544e-007	
R2		0.960		0.601		0.985
Q2		0.833		0.517		0.879
p (CV-ANOVA)		<<0.001		<<0.001		<<0.001
V1	2-Methyl Butanal	0.141	(Z)-3-hexenyl butanoate	0.031	Methyl hexanoate	0.273
V2	1-Pentanol	0.140	1-Methyl-1-butanol	0.028	Dimethyl Sulfone	0.270
V3	2-Butanol	0.126	Ethyl Hexanoate	0.026	Methanthiol	0.207
V4	1-methoxy-4-methyl benzene	0.109	2-Octanol	0.026	Isoamyl Butanoate	0.182
V5	2-Octanone	-0.048	2-Decanone	0.026	(Z)-3-hexenyl butanoate	0.178
V6	2-Hexanol	-0.054	2-Undecanone	0.025	3-Octanone	0.169
V7	Butanoic Acid	-0.076	2-Hexanone	0.025	2-Octanol	0.142
V8	1-Methyl-1-butanol	-0.089	2-Nonanone	0.025	Methyl Butanoate	0.135
V9	Methyl Butanoate	-0.095	2-Hexanol	0.025	1-Heptanol	0.100
V10	Isoamyl Butanoate	-0.109	2-Pentanone	0.025	2-Nonanol	0.038
V11	Methanthiol	-0.114	2-Heptanone	0.025	8-Nonen-2-one	-0.060
V12	2-Ethyl Hexanol	-0.117	Decanoic Acid	0.025	2-Hexanone	-0.075
V13	Methyl hexanoate	-0.135	2-Nonanol	0.025	Acetic Acid	-0.076
V14	3-Octanone	-0.191	2-Octanone	0.025	1-Pentanol	-0.089
V15			2-Heptanol	0.024	Ethyl Butanoate	-0.104
V16			Unidentidied alcohol*	0.024	1-methoxy-4-methyl benzene	-0.117
V17			Octanoic Acid	0.024		
V18			2-Butanone	0.024		
V19			Toluene	0.024		
V20			8-Nonen-2-one	0.024		
V21			Butanoic Acid	0.023		
V22			Hexanoic Acid	0.023		
V23			gamma Caprolactone	0.022		
V24			2-Propanone	0.022		
V25			Ethyl Octanoate	0.022		
V26			Isoamyl Butanoate	0.020		
V27			Ethyl Benzene	0.020		
V28			1-Heptanol	0.020		
V29			Ethyl decanoate	0.020		
V30			1-methoxy-4-methyl benzene	0.019		
V31			Ethyl Butanoate	0.018		
V32			Dimethyl Sulfone	0.018		
V33			Heptyl acetate	0.018		
V34			Acetophenone	0.014		
V35			6-Methyl-5-Hepten-2-one	-0.017		
